# The apoptotic response in HCT116^*BAX-/- *^cancer cells becomes rapidly saturated with increasing expression of a GFP-BAX fusion protein

**DOI:** 10.1186/1471-2407-10-554

**Published:** 2010-10-13

**Authors:** Sheila J Semaan, Robert W Nickells

**Affiliations:** 1 Department of Ophthalmology and Visual Sciences, Medical Sciences Center, 1300 University Ave., University of Wisconsin, School of Medicine and Public Health, Madison, WI, 53706 USA; 2 Department of Biomolecular Chemistry, Medical Sciences Center, 1300 University Ave., University of Wisconsin, School of Medicine and Public Health, Madison, WI, 53706 USA; 3 Department of Reproductive Medicine, Leichtag Biomedical Sciences Building, 9500 Gilman Dr., University of California, San Diego, La Jolla, CA, 92093 USA

## Abstract

**Background:**

Many chemotherapeutic agents promote tumor cell death by activating the intrinsic pathway of apoptosis. Intrinsic apoptosis involves permeabilization of the mitochondrial outer membrane and the release of cytochrome c, a process that is controlled by proteins of the *BCL2 *gene family. Chemoresistance is often associated with abnormalities in concentrations of *BCL2 *family proteins. Although stoichiometirc interactions between anti-apoptotic and BH3-only BCL2 family proteins have been well documented as affecting cell death, the association between changes in BAX concentration and intrinsic apoptosis are poorly understood.

**Methods:**

Exogenous GFP-murine *Bax *fusion constructs were transfected into *BAX*-deficient HCT116 cells. To titrate the expression of the fusion protein, GFP-BAX was cloned into a tetracycline sensitive expression cassette and cotransfected with a plasmid expressing the rtTA transcription factor into HCT116^*BAX-/- *^cells. Linear expression of the fusion gene was induced with doxycycline and monitored by quantitative PCR and immunoblotting. Cell death was assayed by DAPI staining cells after exposure to indomethacin, and scoring nuclei for condensed chromatin and fragmented nuclei.

**Results:**

HCT116^*BAX-/- *^cells were resistant to indomethacin, but susceptibility could be recovered in cells expressing a GFP-BAX fusion protein. Titration of GFP-BAX expression revealed that the concentration of BAX required to induce a saturating apoptosis response from baseline, was rapidly achieved. Increased levels of GFP-BAX were unable to stimulate higher levels of cell death. Examination of GFP-BAX distribution before and after indomethacin treatment indicated that BAX protein did not form aggregates when present at sub-lethal concentrations.

**Conclusion:**

Within the limitations of this experimental system, *BAX*-dependent apoptosis in HCT116 cells exhibits an all-or-none response depending on the level of BAX protein present. The lack of BAX aggregation at sub-saturation levels suggests that the translocation step of BAX activation may be impaired.

## Background

Intrinsic apoptosis is the principal autonomous self-destruct pathway executed by cell somas. Multiple chemotherapeutic agents and radiation are effective by activating this pathway in dividing cells. The critical regulatory step of this pathway involves mitochondrial dysfunction, which occurs through a process controlled by related proteins forming the *BCL2 *gene family. Members of this family share common BCL-2 Homology (BH) domains, which affect their interactions with lipid bilayers and each other [1]. The exact nature of the activation steps are not well known, but pro-apoptotic molecules, such as BAX and BAK, exist in conformationally inactive states in living cells. BAX, for example, is a globular protein in the cytosol. Upon the activation of apoptosis, BAX unfolds to expose critical domains that enable it to translocate and insert to the mitochondrial outer membrane (MOM), and then form multimeric aggregates with itself [2-4]. These aggregates facilitate the release of cytochrome c, either by forming pores in the MOM, or by a direct destabilization of the lipid bilayer. BAX insertion and aggregation is the point of no return in the apoptotic pathway [5]. Once cytochrome c is released, the caspase cascade is activated, and dying cells are subjected to proteolytic breakdown. In addition to the release of cytochrome c, BAX-dependent mitochondrial dysfunction also disrupts electron transport thereby destabilizing the proton gradient across the inner membrane, causing a loss of ATP production and the formation of superoxide anions and other free radicals.

Antagonizing the function of pro-apoptotic BAK and BAX, are anti-apoptotic family members such as BCL2 and BCLX_L_. These proteins generally exist in or at the surface of the MOM, and may interact directly with BAK and BAX to prevent an accidental insertion event at the membrane surface. The balance between pro- and anti-apoptotic molecules is critical, even though some cells reportedly have several fold more anti-apoptotic molecules in abundance than the pro-apoptotic counterparts. At the onset of apoptosis, cells typically activate a several different species of BH3-only proteins, which reportedly preferentially bind to and inactivate the excess numbers of anti-apoptotic BCL2 family proteins. The threshold of BH3-only proteins is apparently set by the concentration of proteins like BCL2, and recent studies indicate that all anti-apoptotic molecules must be sequestered before cell death can occur [6,7]. This purported stoichiometric balance between proteins like BCL2 and BH3-only proteins is the foundation of the modified rheostat model of BCL2 family function originally hypothesized by the late Dr. Stanley Korsmeyer [8,9]. In the revised model, the concentration of BAX is not a critical component of the ability of a cell to adequately neutralize anti-apoptotic BCL2 proteins.

Alterations of the concentrations of BCL2 family proteins can have dramatic effects on cell death. Cells that exhibit resistance to chemotherapy, for example, often show elevated expression of anti-apoptotic proteins [10]. This has led to the development of a new series of potential chemotherapeutic agents that target the process of apoptosis directly, and include small molecules that bind to the hydrophobic groove of anti-apoptotic proteins and antagonize their function [11]. Presumably, these antagonists augment the effects of naturally expressed BH3-only proteins, which normally could not neutralize excess molecules of BCL2 or BCLX_L_.

Even though not directly relevant to the balance between anti-apoptotic and BH3-only BCL2 family proteins, conditions that result in lower than normal BAX expression can also affect cell death. BAX gene mutations that reduce transcription levels, or produce dysfunctional protein, have been correlated with increased resistance of cells in lymphocytic leukemia and colorectal carcinoma, respectively [12-14]. In addition, various neuronal cell-types, which rely solely on BAX for pro-apoptotic activity, become highly resistant to lesions of the nervous system when their BAX levels are reduced [15-18].

The development of successful strategies to influence the balance of BCL2 family proteins in target cells requires a complete understanding of the activation events and interactions between members of this gene family. Our early studies on the role of BAX in neuronal death suggested that apoptosis was critically dependent on the level of this pro-apoptotic protein to execute the cell death pathway, irrespective of the concentration of anti-apoptotic molecules present [18]. In this report, we extend these observations to HCT116 colorectal carcinoma cells, which, like neurons, are completely dependent on BAX for apoptosis. HCT116 cells are susceptible to non-steroidal anti-inflammatory drugs, while HCT116^*BAX -/- *^cells are completely resistant. Susceptibility in *BAX*-deficient cells can be rescued if they express an exogenous BAX gene, but only after a critical level of expression has been achieved. Furthermore, experiments using a GFP-BAX fusion protein, indicate that normal aggregation of BAX is impaired at non-lethal levels, suggesting that the level of BAX is critical for the successful activation of this proapoptotic protein, and not related to overcoming a defined number of anti-apoptotic proteins.

## Methods

### Clones and plasmids

To generate the GFP-BAX fusion protein construct, murine *Bax *was cloned into pEGFP-C3 (Clontech, Palo Alto, CA) by first amplifying *Bax *cDNA using the following primers: 5'ACC CGC CGA GAG GCA GCG (forward) and 5'CAC AGT CCC AGG CAG TGG G (reverse). Nested PCR was used to engineer a *Hind*III and *EcoR*I site onto the *Bax *cDNA for in-frame ligation to the C-terminus of GFP.

To generate constructs in the pTRE-Tight vector (Clontech), GFP-BAX was amplified from the pEGFP-C3 plasmid using 5'GCA TGC GAT AGG TAC CAT GGT GAG CAA GGG CGA GG (forward, includes *Kpn*I site) and 5'GTC GCG TCC TAA GCT TTC AGC CCA TCT TCT TCC (reverse, includes *Hind*III site). The resulting PCR fragment was first blunt-ended and then cut with *Kpn*I. This fragment was ligated into the pTRE-Tight vector cut with *Kpn*I and *Not*I, which had been back-filled with Klenow enzyme. GFP alone was cloned into pTRE-Tight by utilizing the above primers, but cutting the insert with *Kpn*I and *Hind*III to excise GFP alone.

An *S16 *ribosomal protein cDNA was amplified using primers: 5'CAC TGC AAA CGG GGA AAT GG (forward) and 5'TGA GAT GGA CTG TCG GAT GG (reverse). All cDNAs were blunt-end cloned into the *Sma *I site of pBK-CMV (Stratagene, La Jolla, CA).

### RNA isolation and quantitative PCR

Total RNA from transfected HCT116 cells was isolated using Tri-reagent (Molecular Research Center, Cincinnati, OH) and treated extensively with DNase I to eliminate contaminating genomic and transfected DNA. First strand cDNA was synthesized and quantitative PCR was performed using the Applied Biosystems (ABI, Foster City, CA) 7300 real time PCR system and ABI SYBR Green PCR Master Mix as described previously [19]. Control reactions, using samples made without reverse transcriptase were also run to verify amplification was from cDNA templates. Standard curves were generated for each product using cloned cDNAs for GFP-*Bax *and *S16 *ribosomal protein to quantify the abundance of cDNA in each unknown. The qPCR cycling parameters were: 1 cycle of 95°C for 10 min, 40 cycles of 95°C for 15 s and 60°C for 1 min. Data collection was taken at the 60°C annealing/extension phase. In order to ensure the presence of a single product, a dissociation curve was performed after each run and products visualized on ethidium bromide-stained agarose gels. Data were collected from threshold values using the automatic function of the 7300 System Sequence Detection Software program. The primers used to quantify *Bax *cDNA were: 5'TTC ATC CAG GAT CGA GCA GG (forward) and 5' CAT CAG CAA ACA TGT CAG C (reverse). The primers used to quantify *S16 *ribosomal protein cDNA, were the same primers that were used to clone *S16 *above.

### Cell culture

All cells were maintained in a humidified incubator at 37°C with 5% CO_2_. Medium was changed every 2-3 days. Human colorectal cancer cells lacking a functional *BAX *gene, HCT116^*BAX*^^-/- ^[20], were a gift from Dr. Bert Vogelstein. These cells were cultured in McCoy's 5A Medium (Modified) with 25 mM Hepes and L-glutamine (Cambrex, Walkersville, MD), and supplemented with 10% Fetal Bovine Serum (FBS) (certified tetracycline free - Atlanta Biologicals, Atlanta, GA) and 1% penicillin/streptomycin. HCT116 cells were treated with 500 μM indomethacin (Sigma, St. Louis, MO), 24 hrs after transfection (see below) to induce cell death for threshold experiments. Cells attached to the plate and those floating in the medium were harvested 48 hrs after treatment, fixed in 3.7% formaldehyde and 0.5% Igepal CA-630, and DAPI stained. Cells with condensed chromatin and fragmented nuclei were scored as apoptotic as described previously [20].

### Transfections

HCT116 cells were plated at a density of 1 × 10^6 ^cells/well into 6-well plates. All cells were transfected 24 hrs after initial plating. Transfections were performed using the Tfx-50 transfection reagent (Promega) with a 2:1 (transfection reagent: DNA) ratio. Plasmid DNAs used for transfection of HCT116 cells were 1 μg of either pTRE-GFP or pTRE-GFP-BAX co-transfected with 1 μg of pTet-ON (Clontech). The transfection media was replaced after 5 hrs with complete media containing increasing concentrations of doxycycline (DOX - Sigma), from 0 ng/ml to 100 ng/ml. Transfection efficiency for HCT116 cells was evaluated by counting the number of GFP positive cells and calculating the percentage based on the total number of cells. For cell death controls, HCT116 cells were transfected with 2 μg of pGL3-Control, pTRE-GFP-BAX, or pTet-ON.

### Immunoblotting

Immunoblots were performed as described previously [21] with modifications. Lysates from HCT116^*BAX*^^-/- ^cells were used for immunoblots (60 μg/lane). Protein concentration was quantified using the Pierce BCA Protein Assay (Thermo Scientific, Rockford, IL). Rabbit polyclonal antibodies against BAX (B3428) or ACTIN (A2066), were purchased from Sigma and used at 1:2,000 and 1:100 dilutions, respectively. A goat polyclonal antibody against GFP (T-19) (sc-5384) was purchased from Santa Cruz Biotechnology (Santa Cruz, CA) and used at a 1:100 dilution. Goat anti-rabbit secondary antibodies conjugated to alkaline phosphatase were used to visualize antibodies against BAX and ACTIN, while a donkey anti-goat secondary antibody conjugated to horse radish peroxidase was used to visualize the antibody against GFP. Immunoblots were developed with the ECL Western Blotting Detection Reagent from Amersham (Piscataway, New Jersey), scanned on a Storm 860 scanner (Amersham), and band density quantified using ImageQuant v5.2 (Amersham).

### Fluorescent microscopy

Cells were plated, grown, and transfected in tissue culture well slides (Fisher Scientific, Chicago, IL). At the end of each experiment, the cells were fixed as described above, DAPI-stained, and coverslipped. Fluorescent photomicrographs were acquired using a Zeiss Axioplan 2 Imaging microscope with digital camera (Zeiss, Thornwood, NY). Images were pseudo-colored using Zeiss Axiovision Image Analysis software (v4.6). For graphic presentation, these images were imported into Adobe Photoshop, but were not enhanced further.

### Statistical analyses

For data presented as mean ± SEM, assessment for significant differences between groups was performed by Student's *t*-test. A level of *P *≤ 0.05 was used to designate significance.

## Results

### Susceptibility to indomethacin can be restored in HCT116^BAX-/- ^cells by transfection with an exogenous GFP-Bax fusion construct

**Figure 1:**
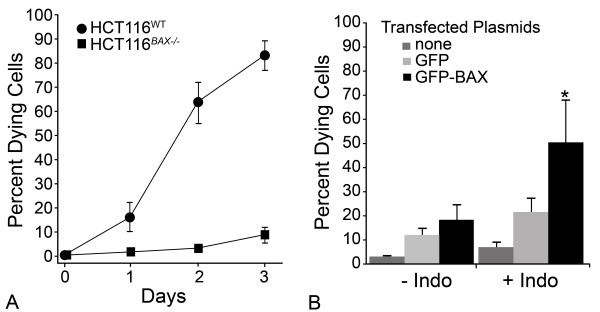
**Transient transfection of GFP-BAX fusion protein into HCT116**^***BAX***^^**-/- **^**cells rescues cell death after Indomethacin treatment**. (A) HCT116^*BAX-/- *^cells are resistant to indomethacin. Line graph showing percentage of apoptotic cells in the medium after exposure of 500 μM indomethacin for wild type (WT) and *BAX*-deficient HCT116 cells over time. (B) Rescue of the cell death phenotype by transfection of an exogenous GFP-*Bax *gene. HCT116^*BAX*^^-/- ^cells were transfected with either no plasmid, pEGFP-C3 (GFP), or pEGFP-C3-BAX (GFP-BAX), which expresses a fusion protein of GFP in phase with the murine *Bax *coding region under the control of the CMV immediate early promoter. Twenty-four hours after transfection, cells were treated with 500 μM indomethacin (+Indo) or vehicle (-Indo). Transfection efficiency was ~60% in each condition, based on the proportion of cells positive for GFP expression. Forty-eight hours after indomethacin treatment, cell death was assessed. Transfection of GFP-BAX into cells that were later treated with indomethacin caused a significant increase in death over GFP transfection and indomethacin-treated cells (*t*-test, *P = 0.0001). Values shown are mean ± SD in both graphs of data collected from 3 independent experiments.

HCT116 cells are susceptible to the non-steroidal anti-inflammatory drug indomethacin, which activates the intrinsic apoptotic program. Unlike other cell types, which utilize both BAX and BAK, this program is solely reliant on BAX function [20], and cells lacking a functional BAX gene are normally resistant (Figure 1A). To determine if susceptibility of HCT116^*BAX-/- *^cells could be restored by the expression of an exogenous BAX protein, we transfected them with a plasmid expressing a GFP-BAX fusion protein under control of the CMV immediate early gene promoter. These cells were treated with indomethacin, 24 hours after transfection, and cell death was assayed after 48 hours. Cells expressing a control plasmid containing only GFP exhibited a modest increase in apoptosis after exposure to indomethacin, suggesting some toxicity associated with the transfection protocol. Cells expressing the GFP-BAX fusion protein, however, exhibited a substantial and significant increase in cell death, but only after treatment with indomethacin (*P *= 0.0001, compared to GFP-transfected cells, Figure 1B). Examination of GFP expressing cells also indicated that these cells accounted for the majority of dying cells in this paradigm (data not shown).

### The cell death response in HCT116^BAX-/- ^cells is rapidly saturated with increasing levels of exogenous GFP-Bax expression

**Figure 2:**
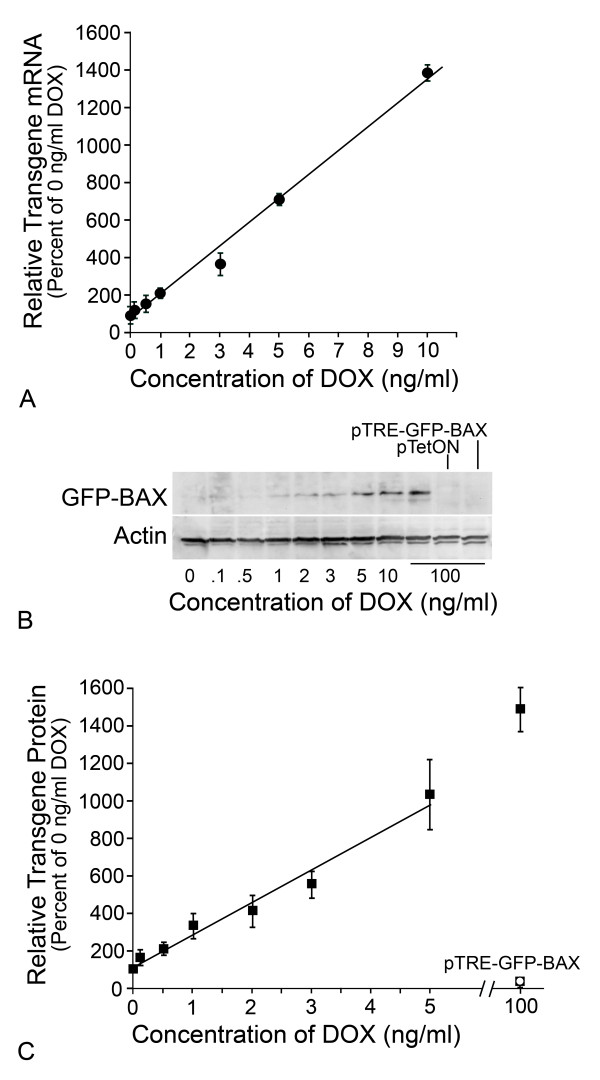
**Titration of GFP-BAX transgene expression in HCT116**^***BAX***^^**-/- **^**cells**. HCT116^*BAX*^^-/- ^cells were co-transfected with pTetON and pTRE-GFP-BAX and exposed to increasing concentrations of DOX in the media. (A) Quantification of transgene transcripts using quantitative (Real-Time) PCR. Data shown were normalized to *S16 *cDNA in each sample and indicated as a percentaqe of the transcript level at 0 ng/ml DOX (mean ± SEM of triplicate samples from 3 separate experiments used for calculations). Levels of transgene mRNA increased linearly with increasing concentrations of DOX up to 10 ng/ml (R^2 ^= 0.991). (B) Immunoblot of transgene expression. GFP-BAX protein expression increased with increasing concentrations of DOX. Low levels of GFP-BAX were detected in cells treated with 0 ng/ml DOX, but not in controls of each plasmid transfected individually (right hand lanes). ACTIN levels in each lane are shown as a loading control. (C) Quantification of GFP-BAX protein expression. Transgene protein levels were quantified, normalized to the amount of ACTIN present in the same sample, and expressed as the percentage of protein levels detected in 0 ng/ml DOX (mean ± SEM of data collected from 5 independent gels). Similar to transcript levels, increasing concentrations of DOX produced a linear increase in transgene protein expression (between 0 and 5 ng/ml DOX, R^2 ^= 0.975).

**Figure 3:**
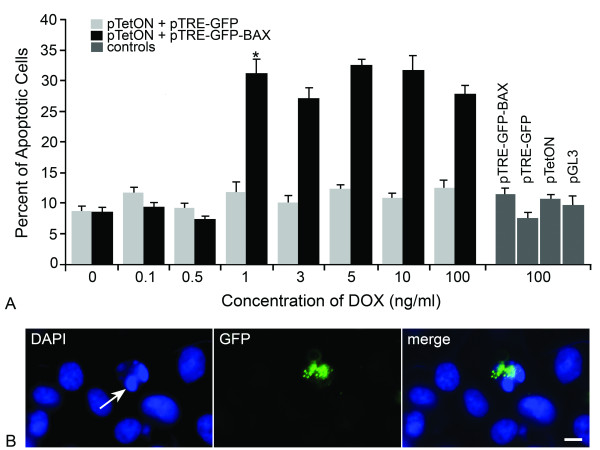
**Indomethacin-induced apoptosis in HCT116**^***Bax***^^**-/- **^**cells becomes rapidly saturated with increasing levels of a GFP-BAX fusion protein**. HCT116^*Bax*^^-/- ^cells were co-transfected with a pTRE-plasmid (carrying either GFP-BAX or GFP) and pTetON, and transgene expression was induced by increasing concentrations of Doxycycline (DOX) in the media. After 24 hrs, cells were treated with indomethacin and cell death assayed 48 hrs later. Cells were also transfected with control plasmids alone and exposed to 100 ng/ml DOX. (A) A histogram showing the percent apoptotic cells 48 hrs after indomethacin treatment at different DOX concentrations (mean ± SEM of 3 separate experiments). Transfection of control vectors alone (pTRE-GFP-BAX, pTRE-GFP, pTetON, and pGL3-Control), or pTetON combined with pTRE-GFP, was observed to elicit 8-10% cell death, indicating that the transfection procedure itself was mildly toxic to these cells. GFP-BAX expression stimulated by 0-0.5 ng/ml DOX was insufficient to cause cell death above these background levels (*P *= 0.40). At the DOX concentration of 1 ng/ml, however, significant cell death was observed (*P = 6.1 × 10^-6^, GFP-BAX vs. GFP). Concentrations of DOX greater than 1 ng/ml did not stimulate apoptosis above this level (*P *= 0.47). (B) A representative photomicrograph of cells transfected with GFP-BAX. DAPI-staining (left panel) showed several cells with normal nuclei and one cell with a fragmented nucleus and condensed chromatin (arrow). GFP imaging of the same field of cells (center) showed one cell expressing the GFP-BAX transgene. GFP-BAX was distributed in a punctate labeling pattern and was localized to the single apoptotic cell in the field (merged image). At this time point after indomethacin treatment, approximately 80% of apoptotic cells were found to be expressing GFP-BAX. Scale bar = 5 μm.

In order to test the association between the level of BAX expression and the ability to activate the apoptotic program, HCT116^*BAX -/- *^cells were co-transfected with a pTRE-plasmid, carrying a tetracycline responsive promoter expressing either GFP-BAX or GFP, and pTetON, which codes for the reverse tetracycline transcription activator, rtTA. Transgene expression was induced for 24 hrs by the presence of doxycycline (DOX) in the media. Both mRNA and protein levels showed a linear increase in abundance between 0-10 ng/ml DOX (Figure 2, R^2 ^= 0.991 for mRNA levels and R^2 ^= 0.975 for protein levels), after which levels increased non-linearly. A low level of GFP-BAX was detected in cells treated with 0 ng/ml DOX, suggesting basal transcription from the pTRE-GFP-BAX vector when co-transfected with pTetON. Cells transfected with pTetON alone, or pTRE-GFP-BAX alone, acted as negative controls for transgene expression. Cells transfected with pTRE-GFP-BAX exhibited only 17.6% of the level of fusion protein detected in doubly transfected cells in 0 ng/ml DOX. After 24 hrs in varying concentrations of DOX, co-transfected cells were treated with indomethacin to induce apoptosis and cell death was assayed after 48 hrs. Transfection of control vectors alone was observed to elicit 8-10% cell death. GFP-BAX expression stimulated by 0-0.5 ng/ml DOX was insufficient to cause cell death above this background level (*P *= 0.40) (Figure 3A). At the DOX concentration of 1 ng/ml, significant cell death was observed (*P *= 6.1 × 10^-6^, GFP-BAX vs. GFP), which did not increase at higher concentrations of DOX (*P *= 0.47) (Figure 3A). Although the level of cell death induced was lower than that observed for HCT116^*BAX*^^+/+ ^cells, it corresponded to the 40% transfection efficiency observed in our experiments. To determine if transgene expressing cells were dying, we verified microscopically that the majority (~80%) of GFP-BAX positive cells also exhibited condensed or fragmented apoptotic nuclei (Figure 3B).

### GFP-BAX aggregation is impaired at sub-lethal levels of expression

**Figure 4:**
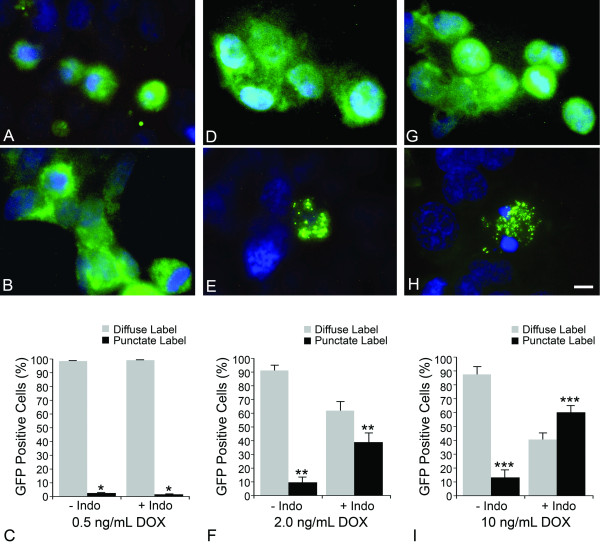
**BAX aggregation is disabled at sub-threshold levels of expression**. All photomicrographs of GFP-BAX staining (with DAPI counterstain) were taken 18 hours after vehicle treatment (A, D, G) or indomethacin treatment (B, E, H). Exposure times were adjusted automatically to maximize detection of GFP-BAX localization. As a consequence, the level of fluorescent intensity of the fusion protein in each condition appears similar, even though protein levels linearly increase with increasing doxycycline (DOX) concentration (see Figure 2). (A-C) HCT116 cells expressing a sub-lethal level of GFP-BAX induced by 0.5 ng/ml DOX. (A) GFP-BAX localization was diffuse in cells not exposed to indomethacin. This pattern of distribution remained unchanged in cells after indomethacin treatment (B). (C) Quantitative analysis of GFP-BAX distribution between conditions. There was no significant increase in punctate labeling of GFP-BAX after indomethacin treatment (*P = 0.195). (D-F) HCT116 cells expressing a supra-lethal level of GFP-BAX induced by 2 ng/ml DOX. Addition of indomethacin caused the redistribution of GFP-BAX from a diffuse pattern (D) to a punctate pattern (E). Quantitative analysis (F) of the distribution of GFP-BAX showed a significant shift in the proportion of cells with punctate labeling (**P = 0.0002). (G-I) HCT116 cells expressing a supra-lethal level of GFP-BAX induced by 10 ng/ml DOX. Similar to cells in (D-F), GFP-BAX showed a significant increase in punctate labeling after indomethacin treatment (***P = 8.9 × 10^-8^). Data for GFP-BAX distribution was collected from a minimum of 100 GFP positive cells per condition and the distribution was scored by 2 masked observers. Scale bar = 5 μm.

The activation of BAX during apoptosis is simplistically divided into four basic steps, including initial activation (conformational change) of cytosolic monomers, translocation to the MOM and insertion into this membrane, secondary recruitment of inactivate BAX monomers by MOM-bound BAX molecules, and aggregation of bound and recruited BAX molecules into oligomers that enable the release of cytochrome c. We investigated if BAX aggregation was altered at non-lethal levels of fusion protein by monitoring the subcellular localization of GFP-BAX in HCT116^*BAX-/- *^cells, before and after, indomethacin treatment (Figure 4). Others, using similar GFP-BAX constructs, had demonstrated that BAX localization changed from a diffuse labeling pattern to more punctate foci, co-localizing with mitochondrial markers, as active protein translocates and aggregates to the MOM [22,23]. In this experimental paradigm, the distribution of GFP-BAX was examined after 18 hours of indomethacin exposure to ensure that cells in an intermediate stage of apoptosis could be examined. Cells expressing GFP-BAX at non-lethal levels exhibited nearly 100% diffuse localization of this fusion protein, with no evidence of significant redistribution of protein to a punctate labeling pattern after indomethacin treatment (*P *= 0.195) (Figure 4A-C). At two levels of lethal GFP-BAX expression, however, cells exhibited a significant increase in punctate labeling after indomethacin treatment (*P *= 0.0002, and *P *= 8.9 × 10^-8^, for 2 ng/ml and 10 ng/ml DOX treatment, respectively) (Figure 4D-I). Thus, the distribution of GFP-BAX after 18 hours correlated with resistance or susceptibility of these cells to indomethacin-induced apoptosis after 48 hours.

## Discussion

Previously, we had observed that the ability of some neurons to execute BAX-dependent cell death was linked to the level of BAX expression in these cells [18]. This dependence on the BAX concentration was independent of the concentration of the principal antagonizing anti-apoptotic protein, BCLX_L_, which was at least 10-fold more abundant in these cells [18,24]. A similar dependence for BAX protein was also observed in *BAX*-deficient HCT116 cells.

The original rheostat hypothesis of BCL2 family protein function [8] predicted that pro- (i.e., BAX) and anti- (i. e., BCLX_L_) apoptotic proteins could form dimers thereby neutralizing each other. In this model, the expression of BH3-only proteins competed with BCLX_L_:BAX dimers and facilitated the release of BAX allowing to become active. This model has been re-examined [9], in light of data showing that heterodimers cannot form under physiologic conditions [25,26]. Instead, the stoichiometric balance between anti-and pro-apoptotic proteins appears to involve proteins like BCLX_L _(anti-apoptotic) and BH3-only proteins (pro-apoptotic) that are expressed or activated in cells stimulated to undergo the apoptotic program. Interaction of anti-apoptotic proteins and BH3-only proteins results in neutralization of the former. This facet of the apoptotic activation cascade does not necessarily result in BAX activation, however, and similarly the concentration of BAX likely does not impact the stoichiometric balance between them. Activation of BAX appears to require an independent interaction with a BH3-containing protein [27-29], which allows it to change conformation in the cytoplasm from a globular monomeric protein to one that is able to translocate and insert into the MOM.

If the dependence for a critical level of BAX protein to activate apoptosis is not associated with the stoichiometric balance between anti-apoptotic and BH3-only proteins, an alternative effect influenced by BAX protein levels may be in the process of BAX activation itself. The impairment of BAX translocation and/or aggregation in cells expressing sub-lethal levels can be interpreted in several ways, depending on the rate-limiting step for BAX activation.

First, the interaction of BAX with a binding partner could be affected by low levels of BAX protein, especially if the binding partner had a reduced affinity for BAX in its globular conformation. Candidates for these binding partners are the BH3-only proteins, which like BIM, have been shown to have higher affinities for anti-apoptotic *BCL2 *family members and can only bind weakly to BAX [30]. Thus, any role they may have in interacting and unfolding cytosolic BAX would be thermodynamically unfavorable in the presence of low BAX concentrations.

Second, the translocation and aggregation of BAX at the MOM may also be concentration dependent. Current models suggest that very few cytosolic BAX molecules actually need to be independently activated (by BH3-only proteins) and translocated, since once they are present in the MOM they act as a sink to capture other BAX molecules, even if the latter are missing their C-terminal mitochondrial targeting domains [4,31]. Under these conditions, aggregation by cooperative binding may rely on passive diffusion of cytosolic BAX, which are only captured at the MOM when they randomly collide with MOM-bound BAX proteins. The lethal concentration of BAX would be defined as the point when random collisions occurred rapidly enough to allow for the timely activation of the apoptotic program; a process that would clearly be affected by the concentration of cytosolic BAX proteins. Eventually, however, one would predict that enough BAX would accumulate in these cells that the apoptotic pathway could be activated. Our titration experiments support this hypothesis since fewer cells show punctate labeling at 18 hrs when treated with 2 ng/mL DOX (Figure 4F), even though they clearly exhibited a saturated apoptotic response at 48 hrs (Figure 3A). Similarly, *Bax*^+/- ^mice, which show neuronal resistance to optic nerve damage at 2 weeks after injury, eventually exhibit dying cells after several months. In contrast, *Bax*^-/- ^cells remain resistant indefinitely [18].

The activating steps of BAX represent a complex process that is not completely understood. Critically, we demonstrated that a very small alteration in the level of BAX was the difference between prolonged cell survival and rapid onset cell death. Thus, the apoptotic switch mechanism involving BCL2 family proteins appears to have two different concentration-dependent components. One, in which a sufficient number of antagonizing BH3-only molecules are required to adequately sequester anti-apoptotic proteins [6,7], and a second, dependent on the level of BAX expression to successfully activate the apoptotic program.

## Conclusion

By titrating BAX expression in *BAX*-deficient HCT116 cells, we demonstrate that the BAX-dependent apoptotic program becomes rapidly saturated with increasing BAX protein. The level of BAX expression appears to affect its ability to form aggregates, which is impaired at sub-lethal concentrations of protein. This marks a second level of control for function of the BCL2 gene family switch that is dependent on the overall cellular concentration of interacting family members.

## Competing interests

The authors declare that they have no competing interests.

## Authors' contributions

SJS participated in the design of the study, performed all the cloning and cell culture studies and data collection as part of her graduate training. RWN participated in study design and supervised the entire project. Both SJS and RWN wrote the manuscript and have approved the final version.

## Pre-publication history

The pre-publication history for this paper can be accessed here:

http://www.biomedcentral.com/1471-2407/10/554/prepub
